# Nitrate as Warden of Nitric Oxide Homeostasis in Mammals

**DOI:** 10.3390/nu17091544

**Published:** 2025-04-30

**Authors:** Barbora Piknova, Ji Won Park, Alan N. Schechter

**Affiliations:** Molecular Medicine Branch, National Institute of Diabetes and Digestive and Kidney Diseases, National Institutes of Health, Bethesda, MD 20852, USA

**Keywords:** nitrate, nitrite, nitric oxide, homeostasis

## Abstract

Homeostasis is the self-regulating processes in cells and organisms designed to maintain stability of the internal environment while adjusting to external changes. To achieve this dynamic stability, internal conditions oscillate within tightly regulated physiological tolerance limits. In mammals, maintaining nitric oxide (NO) availability appears crucial to sustain relatively constant blood flow into all organs and tissues. We hypothesize that NO homeostasis is one of the most important vital processes for warm-blooded animals. It is impossible to conserve the stability of most other vital substances, such as O_2_, CO_2_, blood sugar, pH, and temperature, to name just few, without well-functioning tissue perfusion. NO in mammals is generated either from L-arginine by nitric oxide synthases (NOSs) or by the reduction of nitrate (NO_3_^−^) to nitrite (NO_2_^−^) and NO by several proteins. Here we first discuss the organization of these two NO metabolic pathways, emphasizing that both pathways “cross” and “funnel” unused NO into the overall nitrate-nitrite–NO pathway. This pathway is cyclic, which gives nitrate a unique place in metabolism and predisposes it as a reservoir for NO. Then, we discuss the role of NO homeostasis that, by maintaining organ and tissue perfusion, supports and preserves constancy of other blood-delivered substances. This “governing” role of NO makes even clearer that the existence of NO storage and precursor molecules is necessary, to avoid NO shortages in cases of the precursor’s or storage molecule’s temporary unavailability, to ensure uninterrupted tissue access to NO. We propose that the skeletomuscular system and skin act as nitrate reservoirs assuring NO bioavailability at various external and internal conditions.

## 1. Introduction

The necessary transmission of information among cells and organs appears to have occurred early in the evolution of life. Even the most primitive organisms, from monocellular to cell clusters, and later, multicellular organisms, needed to adapt to changing environments and adjust their metabolism accordingly. There is indication from studies of different existing organisms across kingdoms and phylli, from fungi to mammals, that nitric oxide (NO) is probably one of the most ancient signaling molecules. The formation of guanosine 3′,5′-cyclic monophosphate (cGMP) from NO (the NO/cGMP pathway) appears to be a very old general signaling system [1,2]. NO might even be the first neurotransmitter that existed, managing the basic survival function, such as fear-like behavior, the function it still holds in the animal kingdom [3]. Interestingly, NO function as the blood flow regulator evolved later mainly in reptiles, birds and mammals and only some fishes [4].

While life in general is possible in wide range of outside surroundings, with some bacteria thriving in ice-cold waters or in hot sulfur springs, physiological processes in all organisms can properly function only in a very narrow range of physical conditions. Over eons of their existence, by “trial and error”, whole systems of regulation evolved to “isolate” living organisms from the exterior and to maintain the relative stability of their interior environment. Homeostasis, as it is called in physiology, is a tightly woven net of reactions and processes that allows organisms to live in an ever changing outside environment.

Upholding the control of network of these homeostatic processes, with a multitude of positive and negative feedback loops, is not a small task. It is not unreasonable to suggest the existence of “main control” points/pathways that would govern access to and efficiency of other processes. We believe that, at least in mammals, several of the “main regulators” of homeostasis are processes critically linked to the maintenance of blood flow, mainly by vasodilation and vasoconstriction. Here, we explore in depth the NO cycle organization and reflect on its role in maintaining “general” homeostasis in mammals by controlling vasodilation processes.

## 2. Nitric Oxide Cycle

### 2.1. Short Story of NO Discovery

NO, a diatomic gas molecule, was discovered in 1772 by Joseph Priestley, who is also known for his oxygen discovery in 1774. NO is a colorless, toxic gas, formed during lightning, which is its main natural source. NO’s free radical nature predisposes it to high reactivity and a short lifetime in most biological surroundings. However, this simple molecule is crucial regulator of many physiological processes [5].

Crucial physiological signaling functions of this free radical were described in 1970s by Furchgott, Zawadski, Moncada, Murad and Ignarro. NO was named a molecule of the year in Science in 1992 and in 1998; Furchgott, Ignaro and Murad were awarded the Nobel prize in Physiology or Medicine for their work on NO’s role as a physiological signaling molecule, largely based on its enzymatic synthesis from L-arginine by various nitric oxide synthase (NOS) proteins (for review, see [6]).

Significant formation of NO by the reduction of nitrite by acidic disproportionation in the stomach was described in 1994 [7], and the alternative pathway of NO formation from nitrite and nitrate started to gain more attention. Around the same time, one group observed nitrite reduction to NO in ischemic heart under acidotic and hypoxic conditions [8,9]. In the early 2000s, the reduction of nitrite to NO in the blood by deoxyhemoglobin was described [10,11,12,13], and the general role of deoxyheme proteins as nitrite reductases was proposed [14], while nitrite effects on vasculature were extensively studied (for reviews, see [13,15]). Interestingly, some nitrite effects might also be unrelated to NO formation [16].

The formation of nitrite, the direct NO precursor, from dietary nitrate in the oral cavity by symbiotic bacteria was also described in the late 1990s [17]. This put nitrate in the spotlight for the first time and highlighted the importance of a functional microbiome for maintaining good health [18]. Since then, evidence for many health benefits of this ion in healthy people and in patients with several diseases is still growing [19,20,21,22,23]. The discovery of mammalian nitrate reductases in 2010 showed that xanthine oxidoreductase (XOR) in mammalian cells, especially in the liver, can reduce nitrate to nitrite, albeit at slower rate than bacteria [24]. However, the general assumption is that mammals still need their microbiome to help to supply sufficient nitrate reduction capacity and for the reaction to happen in the right compartments [18,25,26,27]. However, these findings led to the idea that nitrate, as a precursor of nitrite and NO, is an ultimate candidate as an NO storage molecule. Indeed, in 2015, we showed that skeletal muscle and, later, also skin and the skeleton, the largest organs in mammalian bodies, retain elevated concentrations of nitrate and can serve as mammalian nitrate reservoirs for times when dietary nitrate is unavailable or local needs for increasing NO production occur [28,29].

### 2.2. Organization of the NO Pathway with Emphasis on Its Cyclicity and Self-Sustaining Nature

**Figure 1:**
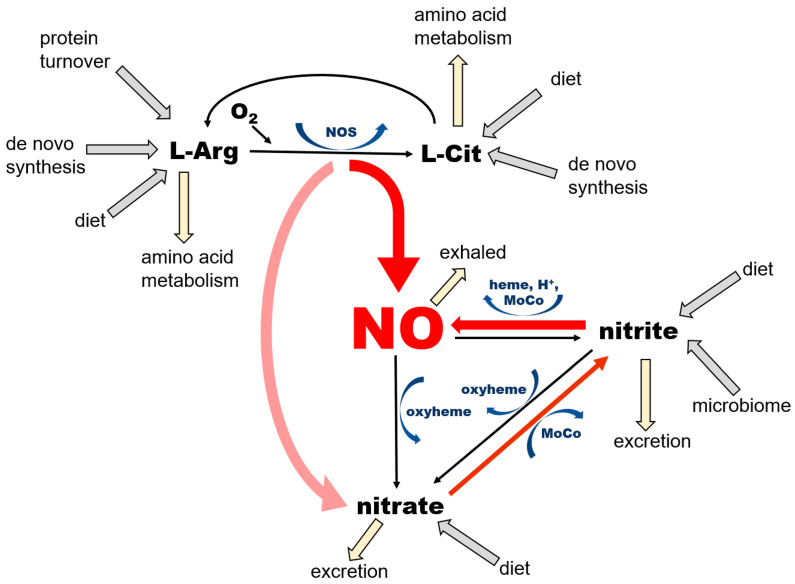
Current understanding of the main pathways of nitric oxide (NO) formation.

Thus, as just summarized, in mammals NO is formed by 2 pathways that ensure its availability at variable physiological conditions, especially with variable oxygen accessibility (Figure 1).

NO is a product of enzymatic conversion of L-arginine (L-Arg) to L-citrulline (L-Cit) by three nitric oxide synthase (NOS) enzymes. This reaction requires the presence of oxygen and several co-factors for NOS, some of which (such as tetrahydrobiopherine, BH_4_) are highly susceptible to oxidation. There are two possible outcomes of this reaction—NO and nitrate—depending on kinetics of NO release from heme, which differs among the NOS isoforms. According to [30], eNOS produces mostly NO (“productive cycle”), and more than 60% of NO synthetized by nNOS is released from heme as nitrate (the “futile cycle”). The nitrate cycle operates without the presence of oxygen and relies on the nonenzymatic reduction of nitrate to nitrite by nitrate reductases—proteins containing molybdopterin motive (MoCo-proteins) that are either of mammalian origin, including xanthine oxidoreductase (XOR), aldehyde oxidase (AO), sulfite oxidase (SO), mitochondrial amidoxime reducing component 1 (mARC) or of bacterial origin (in the saliva, gut, and skin microbiome). The next step, leading to the formation of NO by the reduction of nitrite, is also nonenzymatic and is accomplished by MoCo-proteins, ferrous 5-coordinated heme-proteins (deoxyHb, deoxyMb, cytochromes) or acidic disproportionation at low pH (gastric juices, ischemic acidosis and alike). Unused NO and nitrite can be oxidized back to nitrate by the oxyheme of several usually abundant proteins (oxyHb, oxyMb). The oxidation of NO to nitrite in plasma by ceruloplasmin had been also proposed [31].

NO is a product of the conversion of L-arginine to L-citrulline by the three variants of the NOS enzyme, which are ubiquitous in all tissues, with their highest levels described in endothelial cells (eNOS, NOS 3) and the brain (nNOS, NOS 1). NNOS is also an important part of dystrophin complex in skeletal muscle cells. This pathway requires oxygen and several cofactors—nicotinamide adenine dinucleotide phosphate (NADPH), tetrahydrobiopterin (BH_4_), flavin adenine dinucleotide (FAD), flavin mononucleotide (FMN), heme, and a calcium–calmodulin complex. NOS enzymes, particularly the nNOS isoform, are also known to oxidize formed NO directly to nitrate during its so-called “futile” cycle; therefore, NOS functions also as NO oxidase (for review see [32]).

NO is also the product of the chain reduction of nitrate. Nitrate enters the body mainly via the diet, especially vegetables, with beets and green leafy vegetables being the best natural sources [33,34]. After absorption from stomach to bloodstream, ~25% of ingested nitrate enters salivary glands, where a small portion of it (~5%) is reduced to nitrite by salivary bacteria [27]. Afterwards, as noted above, nitrite-rich saliva is swallowed and enters the stomach. Here, due to highly acidic environment, some of the nitrite load is converted to NO by the reaction of acidic disproportionation and diffuses directly to the bloodstream via the stomach wall [7], and the remaining nitrite re-enters the bloodstream and is carried into tissues. Nitrite is then reduced to NO locally in tissues by either xanthine oxidoreductase (XOR) or deoxy-ferrous heme [18,26].

Nitrate itself is also absorbed from the stomach and passed into the bloodstream, but the majority of it, up to ~75%, is excreted to urine by kidneys [35]. A portion of nitrate that is not processed to nitrite or excreted enters cells via sialin [36,37,38,39] or CLC transporters [38,40], with both transporters ubiquitously distributed on cell surface of many organs. With the exception of its reduction to nitrite, human cells are unable metabolize nitrate [18], which predisposes this ion to be an excellent inert NO storage molecule, with the largest accumulation found in skeletal muscle, skin, and the skeleton [29]. The nitrate–nitrite–NO reductive pathway does not require oxygen, and, in fact, oxygen acts as its inhibitor, due to its inhibitory effects on bacterial and mammalian nitrate and nitrite reductases. This pathway is activated by a low pH, which allows the body to increase NO supply quickly and contributes to exercise-induced functional hyperemia in skeletal muscle [41] and heat-induced hyperemia in skin [42], both due in part to the lowering of pH.

**Figure 2:**
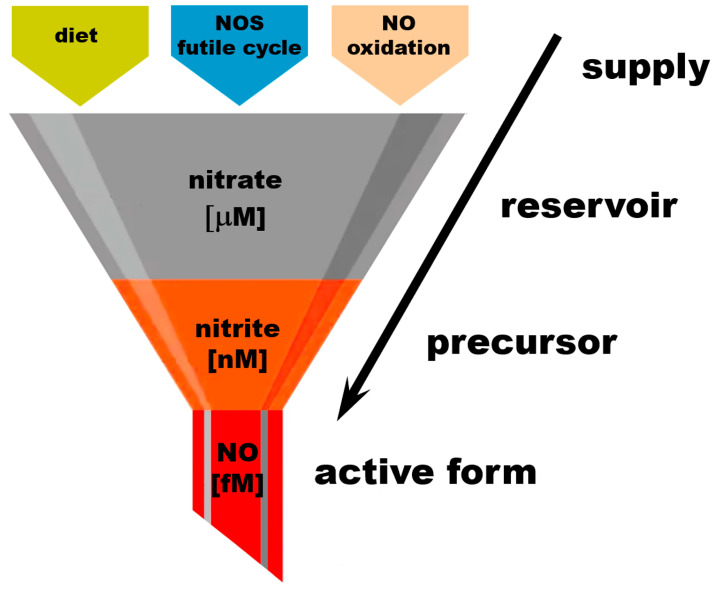
Nitrate as an “NO reservoir” molecule.

The complete NO cycle, combining both NO formation branches (from L-arginine and nitrate), together with intake routes, excretion, and side pathways, is also shown in Figure 1. Notice that NO is not the “main product of interest” in the NOS-dependent pathway, because both L-arginine and L-citrulline are involved in extremely complicated networks of pathways. NO and nitrate produced by the NOS pathway feed the nitrate reduction cycle, where NO production and the conservation of nitrate and nitrite appear to be the main goals. Two out of three reactions of this cycle “run” both ways—reduction and oxidation—which is particularly important from the point of view of the self-renewal and sustainability of the whole cycle. Nitrate in this pathway originates not only from the direct, dietary source as an inorganic nitrate ion, but also from dietary L-arginine and L-citrulline, where the so-called “futile NOS cycle” generates more nitrate than NO. The NOS “shortcut” directly to nitrate is probably an evolutionary way to prevent the synthesis and accumulation of large amounts of unwanted and potentially harmful free radicals, such as NO itself and potentially other NO-derived oxidating agents, such as peroxinitrite (ONOO^-^) [43]. Nitrate itself, as a relatively inert substance, is either stored in tissue reservoirs—mostly in skeletal muscle, skin and bone (see below)—or reduced on demand to nitrite and NO in the nitrate reduction cycle. One should appreciate the fact that even nitrite, which is still considerably less reactive than NO, is not present in large quantities (Figure 2) but rather oxidized and stored as nitrate, which prevents the oxidation of ferrous heme proteins and potential increase in free radical levels.

The amounts of active “free NO” necessary for normal physiological processes are estimated to be in the high fM range and are usually not measured directly due to the short half-life of NO in a biological millieu. Free NO in the presence of oxygen is quickly oxidized to nitrate by many heme proteins. Nitrite concentrations in tissues were measured in the low–mid-nM, with nitrite being significantly less reactive than NO itself but still susceptible to oxidation to nitrate by heme proteins. Almost all circulating nitrite originates from the reduction of nitrate; only small amounts are ingested from diet. At baseline conditions, nitrate concentrations reach the mid-mM range. The main nitrate sources are diet, the NOS futile cycle, and the oxidation of NO. Increasing dietary nitrate intake leads to significant increase in nitrate levels detected in the body, to the high-mM range. This “gradient” of nitrate–nitrite–NO concentrations can be represented as a “funnel” supplying nitrate from its sources to the reservoirs in tissues and to its final active product, NO, through its immediate precursor form, nitrite.

Thus, the old proverb “all roads lead to Rome” becomes true when talking about the NO cycle. The cyclicity of this pathway is the safeguard Nature provided to prevent oxidative damage from excess NO and its adducts, and to assure quick and uninterrupted access to NO by storing abundant amounts of inert nitrate. Remarkably, a literature search for mammalian metabolic pathways involving nitrate shows only one result, nitrate reduction into NO, which further confirms and underlines the unique purpose of nitrate in life-sustaining processes in mammals.

### 2.3. Nitrate as an Exclusive Storage Molecule of NO Cycle

As emphasized already, it is impossible to store NO in its active form due to its free radical nature leading to a half-life time range from a few milliseconds to less than a second in the biological milieu. It must be stored in some less reactive form; theoretically, two main candidates are to be considered—L-arginine and nitrate.

Interestingly, plasma concentrations of both L-arginine and nitrate are in comparable concentration ranges. L-arginine baseline plasma levels in the Framingham offspring cohort were 77.46 ± 18.2 mM [44], while nitrate ranged from 29.3 to 53 mM with a median of 38.4 mM [45]. It was shown that L-arginine levels are also age- and sex-dependent, ranging from 72.4 ± 6.7 μM in young women to 113.7 ± 19.8 μM in elderly men [46]. NO formation from L-arginine by NOS is highly dependent on the redox state of tissue, as some of the NOS cofactors, in particular tetrahydrobioptein (BH_4_), are highly susceptible to oxidation, and oxidized, or “uncoupled”, NOS enzymes synthetize superoxide instead of NO, further increasing oxidative stress. Intracellular levels of L-arginine in bovine aortic endothelial cell (BAEC) cultures can reach 840 ± 90 μM [47], but it seems that in some cases, eNOS, which is a membrane-bound protein, needs to reach to the levels of the extracellular L-arginine supply, despite its high intracellular concentration [48,49]. In addition, L-arginine and L-citrulline are also actively involved in many other physiological pathways on their own [50].

Nitrate concentration in the mammalian body is extremely dependent on diet, which is one of the main sources of this anion. Vegetables, in particular beets and green leafy vegetables, quickly elevate baseline blood nitrate concentration several folds—from 61 ± 6.5 μM to 597.6 ± 23 μM [51]—as an example of the magnitude of the effect, and it is strongly affected by history of dietary nitrate supply [52]. Due to the nature of nitrate distribution—through the bloodstream—and the ubiquitous presence of sialin and CLC transporters on cell membranes, it is not a surprise that nitrate is present in various quantities in all organs. However, when compared at baseline, skeletal muscle is still the organ with the relatively highest concentration when compared to other tissues, even when differences between individual muscle groups persist [53]. The reason for this initial baseline nitrate accumulation in muscle has not been studied in detail, but some possibilities emerge. High contents of functional nNOS, especially the isoform nNOSm, associated with submembrane dystrophin complex [54,55] and well known for its “futile” cycle resulting in the formation of nitrate instead of NO [56], points to the high importance of an intrinsic source of nitrate in this tissue. The functionality of nNOSm, acting as a structural protein and mechanoreceptor [57,58], is critical for skeletal muscle health, and its dissociation from the dystrophin complex is observed in Duchene and Becker muscular dystrophies [59,60], with some evidence that dietary nitrate is able to partially compensate for the loss of NOS functionality [61]. Interestingly, the magnitude of dietary contribution into skeletal muscle nitrate stores depends on the dietary history of this anion. Increasing its supply, either as bolus ingestion (human [62,63] or pig [29] studies) or addition into supply of drinking water for rodents [41,64] leads to significant increase in nitrate levels in skeletal muscle, plasma and other organs. Interestingly, after the skeletal muscle nitrate stock was depleted by a low-nitrate diet, providing high-nitrate drinking water led to an increase in muscle nitrate levels to an extent significantly greater than in animals not subject to nitrate deprivation [52]. As of today, due to its extreme dependence on diet and its history, the range of “normal” nitrate levels in healthy people is still unknown.

Nonetheless, this baseline nitrate “excess” is a factor to consider when thinking about skeletal muscle as nitrate reservoir. Skeletal muscle is also the largest organ/tissue in the body, so even a modest difference in concentration leads to enough “extra nitrate” to supply other organs, which predisposes skeletal muscle to being a “nitrate reservoir”, even before considering any further nitrate supplementation by modified diet [29,63]. As previously shown, dietary nitrate distributes easily into many tissues of several animal species—mice, rats, pigs, and humans [29,62,64,65].

The kinetics and distribution of bolus ingestions of beet root juice, supplying about 16 mM of ^15^N-labeled nitrate ingested by healthy volunteers, was followed for 24 h in plasma, urine, saliva and skeletal muscle [66]. Interestingly, an initial dramatic increase in nitrate in all samples was followed by a return to the baseline, with values for skeletal muscle staying slightly above baseline even after 24 h. An alternative idea to explore, based on the total amount of nitrate in muscle tissue, is that, at certain conditions, baseline concentrations of nitrate in different organs and tissues could fall to similar levels—as seen in the case of a nitrate-deprived diet [52], or as could be the case in some metabolic diseases caused by/associated with disturbances in NO production [67]. As skeletal muscle and skin are the largest organs in body, the total amount of nitrate stored would still exceed the amount stored in other organs, and this amount would be available for all other organs via the bloodstream. The nitrate concentration gradient between blood and muscle could result from different speeds of nitrate reduction and its use by different organs. We believe that liver, with a substantial amount of XOR, would become a prime site for nitrate reduction into nitrite, as well as at sites containing certain bacteria. In this hypothesis, initial nitrate accumulation in skeletal muscle above levels observed in other organs is not a necessary precondition for skeletal muscle functioning as a nitrate reservoir.

Based on the combination of measured nitrate and nitrite concentrations in various tissues of the Yorkshire pig and the known mass of various human organs and tissues, we estimated that in a 70 kg human, there would be only about 400–500 mg of nitrate and about 3–4 mg of nitrite at baseline. These values increased considerably by ingesting a single bolus of 0.15 mmol nitrate/kg, to 1–1.3 g of nitrate and 3–5 mg of nitrite [29]. Considering that the total nitrogen content of a 70 kg human body is about 3% (or 2.1 kg), most of which is contained in nucleic acids and proteins, free nitrate and nitrite ions represent only 0.02% and 0.0002% of total body weight, respectively, yet appear to be very important in maintaining overall constancy of NO availability and, thus, importantly, blood flow.

### 2.4. NO and Blood Flow Control as a Critical Factor for Maintaining General Homeostasis in Mammals

Warm-blooded organisms can only function properly in a very narrow range of internal parameters—such as temperature, oxygenation, ionic strength, nutrient levels, and many others. The process of maintaining these parameters within a viable/optimal range is called homeostasis. In general, homeostasis can be seen as a complicated network of processes and reactions that maintain individual parameters oscillating around the desired ideal steady state. Due to the complicated relationships among the individual steady states and their inter-relations, it is almost impossible to clearly decipher their relationships and influences on each other. However, when a very simplified engineering approach is used, it becomes clear that there must be several “nodes” or “switches” for distinct parts, or perhaps even for the whole network, to be regulated.

Borrowing the engineering point of view, mechanically, the mammalian body is composed of distinct organs and tissues that are interconnected by “distribution” roads, namely circulatory, lymphatic and nervous systems. Actions on any of these three systems will affect the state of all organs, so when looking for homeostatic “switches”, it is likely to be processes governing the functioning of “distribution routes”. Interestingly, NO has an important role in all three systems, by regulating blood [68] and lymph vessels tone [69,70] and as a retrograde neurotransmitter in the brain, regulating the brain blood flow [71,72,73]. As one would expect, as these three processes are connected to the distribution of nutrition, oxygen, signaling molecules and waste removal (blood flow), neurotransmission, and immune defense (lymph and blood flow), all of these are critical to the survival of the organism.

A well-functioning blood flow and cerebral blood flow (CBF) are essential to the preservation of stable settings of vital parameters, such as thermoregulation, oxygenation, pH, flow of nutrients (glucose and others to supply energy), and the maintenance of correct osmolarity in body. The conservation of CBF assures sufficient supply of oxygen and energy and provides an additional layer of regulation of homeostatic settings by hormones and other control substances excreted by the brain. NO, as the main vasodilator, directly controls blood flow and, therefore, the distribution of vital substances carried by the bloodstream. As such, NO is one of the main “switches” for homeostasis regulation. Under normal conditions, a large part of the required NO originates in the NOS productive cycle; however, NO is a very short-lived reactive molecule, and some NOS cofactors are susceptible to oxidation, which renders NOS unfunctional. Nitrate, besides its reduction to nitrite, is an inert molecule in the mammalian body and is therefore the ideal reservoir, or storage molecule, for NO. To some extent, nitrate is found in all organs, with significantly higher levels observed in musculoskeletal system and skin, which are nitrate reservoirs. These two reservoirs can supply nitrate to other tissues via an extensive network of blood vessels that they contain and are resupplied by the same vascular network under favorable conditions of nitrate from diet. Skeletal muscle, due to its high content of nNOS, is also supplied locally with nitrate from the nNOS futile cycle and NO oxidation.

**Figure 3:**
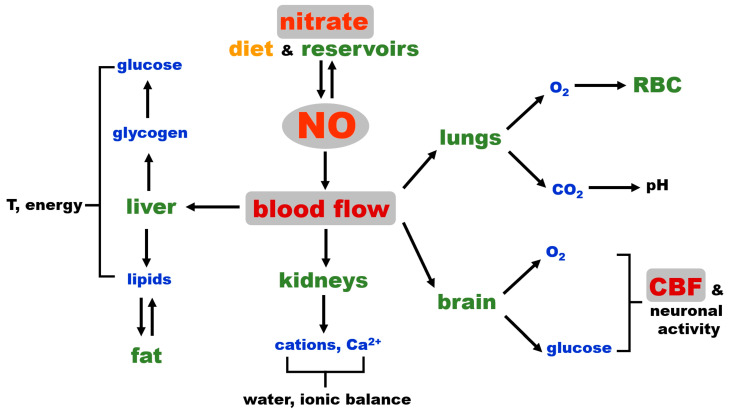
Schematic representation of the NO connection to mammalian homeostasis. CBF: cerebral blood flow, RBC: red blood cells.

Figure 3 shows the dominant role of blood flow in regulating global homeostasis and NO as a key factor that governs blood flow by regulating vascular tone. Critical processes, such as oxygenation, the maintenance of pH, electrolyte balance, osmolarity, water retention, thermoregulation, nutrient distribution and waste removal are directly dependent on the degree of tissue perfusion by blood, while immune responses also depend on blood and lymph flow. NO, needed to increase blood flow (tissue perfusion), can either originate from NOS enzymes (likely eNOS in vascular wall) and be used immediately for vasorelaxation on the site of production or is provided by the in situ reduction of nitrite coming from the tissue nitrate reservoir. The latter possibility could be a safeguard put in place in case of eNOS deficiency at that site or as supplemental NO, contributing to attaining fully functional hyperemia faster, such as during intense exercise when NO increases muscle blood flow and acts as a potent bronchodilator.

Interestingly, the NO bronchodilatory effect is almost never mentioned when considering NO’s role in exercise-induced functional hyperemia in muscle in healthy people, but this link might be a necessary part of increased tissue oxygenation under some extreme conditions or in diseases. In certain studies, inhaled NO improved exercise capacity in patients with precapillary pulmonary hypertension, and in chronic obstructive pulmonary disease (COPD) patients, the inhalation of NO during exercise moderately reduced pulmonary hypertension [74,75]. In another study, in patients with idiopathic pulmonary fibrosis (IPF), inhaled NO reduced mean pulmonary arterial pressure at rest and exercisem and the authors concluded that “in IPF, some endothelium-derived signaling molecules may modulate the development of pulmonary hypertension during exercise, and that the administration of inhaled NO reduces pulmonary vascular resistance without disturbing gas exchange”, which is consistent with the presence of the nitrite/nitrate pathway [76]. NO activity in the respiratory system was also documented for inhaled NO or nebulized nitrite [77,78] and for IV injections of nitrite in rats [79]. Inorganic nitrite distributed by IV supplementation also improved exercise tolerance in heart failure patients with preserved ejection fraction [80]. We believe that more research is needed to understand this important and, so far, under-estimated link between pulmonary function and blood circulation.

While NO vasodilatory control over the general body vasculature is crucial for overall homeostasis, as discussed above, in addition, we would like to emphasize that NO is also among the important, if not the major, regulators of cerebrovascular blood flow (CBF) and neurovascular coupling [73]. This gives NO an additional layer of homeostasis control on a “higher level”, indirectly influencing the level of hormones released by the brain, since the brain cannot function without constant oxygen and glucose supply for extended periods of time, and permanent brain tissue damage occurs minutes after the cessation of blood flow, such as in the case of cardiac arrest or in severe hypoglycemia [73,81,82]. While CBF is still a part of general blood flow, we believe that its high position in the control hierarchy deserves a special reminder.

**Figure 4:**
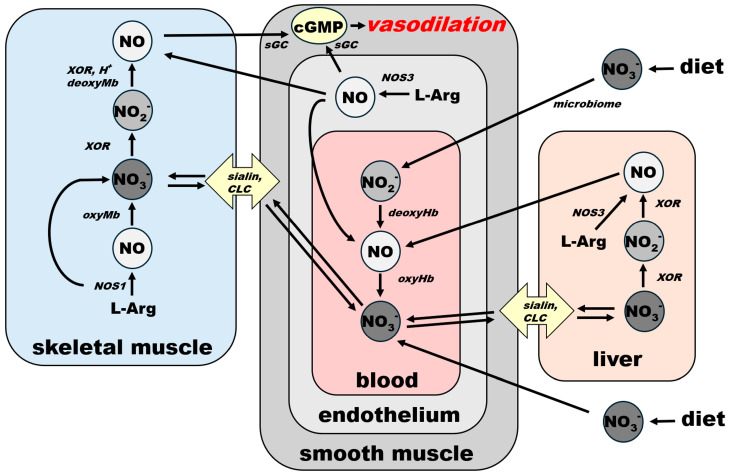
Schematic representation of spatial organization of NO metabolic pathways in skeletal muscle, bloodstream and liver and their connection of NO-cGMP signaling cascade in smooth muscle leading to systemic vasodilation. For clarity, NO effects that are not directly connected with vasodilation—platelet aggregation prevention, neurotransmission, antiseptic effects—are omitted from this scheme. Effects that depend on blood flow regulation (see Figure 3) appear to follow this general scheme. sGC: soluble guanylate cyclase, cGMP: cyclic GMP, CLC: chloride channels and transporters, NOS1: nNOS, NOS3: eNOS, XOR: xanthine oxidoreductase.

Connections and common paths to the final NO-cGMP cascade of NO from both sources—NOS and nitrate—are underlined in Figure 4 via the schematic spatial organization of the NO pathway in several organs. Blood vessel vasodilatory statuses, directly responsible for homeostatic responses, are listed in Figure 3. However, it is also important to be aware that vasodilation is only half of the equation when it comes to general homeostasis. The body, as a closed system, has a limited supply of blood. Therefore, an equally well-controlled system providing shunts or restricting blood flow in less-used parts or organs must exist at the same time. It is out of the scope of this work to explore the endothelin-led vasoconstriction system, but we have to keep in mind that NO and endothelin are equal players that must react in concert to maintain homeostasis.

## 3. Conclusions

In this review, we have tried to show that the two recently characterized pathways for NO formation—by oxidation based on the NOS isoforms and by reduction from nitrate (or nitrite)—completely overlap and will likely compensate for each other. In this context, especially in view of these metabolic interconversions, the homeostatic control of the mammalian (in particular, human) body via the NO regulation of the vascular circulation can be considered to be the most crucial underpinning of the response of the body to environmental variations.

We believe that considering NO (as well as nitrate and nitrite) in this perspective involving the transfer of these species among organs is much more productive than studying its formation and consumption in individual tissues and organs. A system-driven approach, instead of an organ-centered one, facilitates the development of a potential understanding of the contributions of these species to specific pathophysiologies and, most importantly, of their use for therapeutic purposes.

## Abbreviations

The following abbreviations are used in this manuscript:

AO	aldehyde oxidase
BH4	tetrahydrobiopterin
BAEC	bovine aortic endothelial cells
CBF	cerebral blood flow
cGMP	guanosine 3′,5′-cyclic monophosphate
CLC	chloride channels and transporters
COPD	chronic pulmonary obstructive disease
eNOS/NOS3	endothelial nitric oxide synthase
nNOS/NOS1	neuronal nitric oxide synthase
FAD	flavin adenine dinucleotide
FMN	flavin mononucleotide
IPF	idiopathic pulmonary fibrosis
mARC	mitochondrial amidoxime reducing component
MoCo proteins	molybdopterin motive containing proteins
NADPH	nicotinamide adenine dinucleotide phosphate
NO	nitric oxide
NO_2_^−^	nitrite
NO_3_^−^	nitrate
NOS	nitric oxide synthase
ONOO	peroxinitrite
RBC	red blood cell
sGC	soluble guanylate cyclase
SO	sulfite oxidase
XOR	xanthine oxidoreductase
